# Combination model of neutrophil to high-density lipoprotein ratio and system inflammation response index is more valuable for predicting peripheral arterial disease in type 2 diabetic patients: A cross-sectional study

**DOI:** 10.3389/fendo.2023.1100453

**Published:** 2023-02-16

**Authors:** Yi Song, Ying Zhao, Yan Shu, Liyin Zhang, Wenzhuo Cheng, Li Wang, Meng Shu, Baorui Xue, Ruonan Wang, Ziyun Feng, Yao Yin, Fangyang Yu, Si Jin

**Affiliations:** ^1^ Department of Endocrinology, Institute of Geriatric Medicine, Liyuan Hospital, Tongji Medical College, Huazhong University of Science and Technology, Wuhan, China; ^2^ Geriatric Medicine Center, Key Laboratory of Endocrine Gland Diseases of Zhejiang Province, Department of Endocrinology, Zhejiang Provincial People’s Hospital, Affiliated People’s Hospital, Hangzhou Medical College, Hangzhou, China

**Keywords:** type 2 diabetes, peripheral artery disease, inflammation, lipid metabolism, biomarker

## Abstract

**Background:**

Neutrophil/high-density lipoprotein (HDL) ratio (NHR), monocyte/HDL ratio (MHR), lymphocyte/HDL ratio (LHR), platelet/HDL ratio (PHR), systemic immune-inflammation index (SII), system inflammation response index (SIRI), and aggregate index of systemic inflammation (AISI) have been recently investigated as novel inflammatory markers. Herein, the correlation was investigated between these inflammatory biomarkers and peripheral arterial disease (PAD) in type 2 diabetes mellitus (T2DM) patients.

**Methods:**

In this retrospective observational study, the hematological parameter data of 216 T2DM patients without PAD (T2DM-WPAD) and 218 T2DM patients with PAD (T2DM-PAD) at Fontaine stages II, III or IV stage had been collected. Differences in NHR, MHR, LHR, PHR, SII, SIRI, and AISI were analyzed, and receiver operating characteristic (ROC) curves were used to analyze the diagnostic potential of these parameters.

**Results:**

The levels of NHR, MHR, PHR, SII, SIRI and AISI in T2DM-PAD patients were significantly higher than in T2DM-WPAD patients (*P* < 0.001). They were correlated with disease severity. Further, multifactorial logistic regression analyses showed that higher NHR, MHR, PHR, SII, SIRI, and AISI might be independent risk factors for T2DM-PAD (*P* < 0.001). The areas under the curve (AUCs) of the NHR, MHR, PHR, SII, SIRI, and AISI for T2DM-PAD patients was 0.703, 0.685, 0.606, 0.648, 0.711, and 0.670, respectively. The AUC of the NHR and SIRI combined model was 0.733.

**Conclusion:**

The levels of NHR, MHR, PHR, SII, SIRI, and AISI were higher in T2DM-PAD patients, and they were independently linked with its clinical severity. The combination model of NHR and SIRI was most valuable for predicting T2DM – PAD.

## Introduction

Patients with T2DM and PAD have a cardiovascular mortality risk five times higher than patients with only one disease (1, 2). Therefore, early recognition and intervention of PAD in diabetic patients are necessary to lower the risk of major adverse limb events (MALEs) (3). The ankle-brachial index (ABI) is currently recommended as the primary screening tool for PAD in diabetic patients and those with multiple risk factors (4). Due to the low sensitivity of the ABI for early-stage PAD identification, finding new biomarkers that can identify PAD in diabetic individuals at an early stage is urgent.

Recent research has suggested that inflammation and lipid metabolism play a significant role in PAD pathogenesis (5, 6). Besides its capacity to transport cholesterol in the opposite direction, high-density lipoprotein cholesterol (HDL-C) has various protective properties, such as those related to infection, inflammation, antioxidants, and thrombosis (7). Obtaining the numbers of neutrophils, monocytes, lymphocytes, and platelets is inexpensive and easy *via* complete blood count, which are altered when an organism is inflamed. Furthermore, new hematological parameters related to HDL-C and complete blood cells, such as NHR (8), MHR (9), LHR (10), PHR (11), SII (12), SIRI (13, 14), and AISI (15) have been proposed as novel inflammatory biomarkers.

Relevant studies with single or two combined indicators in populations with diabetes or PAD are currently available (16–24). However, until now, no data has been found about how NHR, MHR, LHR, PHR, SII, SIRI, and AISI are linked to T2DM-PAD patients. Herein, the connection was explored between these inflammatory biomarkers and T2DM-PAD patients.

## Materials and methods

### Study population

Gender-matched individuals with T2DM were consecutively recruited from the Department of Endocrinology and Metabolism of the Liyuan Hospital affiliated to Tongji Medical College, Huazhong University of Science and Technology (Wuhan, China), from 1 June 2020 to 31 September 2022. The inclusion criteria are outlined in **Table 1**. Based on the T2DM international criteria (ADA) (25), T2DM was defined as a fasting plasma glucose level ≥ 7.0 mmol/L and/or 2-h plasma glucose ≥11.1 mmol/L during oral glucose tolerance tests (OGTT) and/or glycosylated hemoglobin (HbA1c) level ≥ 6.5%. Each patient included in the study was evaluated for a history of PAD symptoms or a verified diagnosis of PAD using the criteria established by the *Ad Hoc* Committee on Reporting Standards of the Society for Vascular Surgery and the International Society of Cardiovascular Surgery (26, 27). The ABI was measured in patients with PAD-like symptoms, and the physician evaluated the patient’s lower extremities using arterial Doppler-enhanced ultrasonography if they had symptoms in both legs. Patients who had an ABI > 0.90 but showed no symptoms of PAD were not further tested for the disease.

**Table 1:** Inclusion and exclusion criteria.

Inclusion criteria	Exclusion criteria
a) 18-79 years b) Confirmed diagnosis of type 2 diabetes mellitus at the time of admission c) Complete blood count parameters and lipid profile data are available	a) Undefined type of diabetes or clinical suspicion of non-type 2 diabetes mellitus b) Acute complications of diabetes mellitus (such as diabetic ketoacidosis, hyperglycemia hyperosmotic state, and lactic acidosis) c) Chronic kidney disease with eGFR below 60 mL/min (according to the CKD-EPI equation) d) Confirmed liver cirrhosis with Child–Pugh C functional impairment e) Leukocytosis (> 10 × 10^9^ cells/L), leukopenia (< 4 × 10^9^ cells/L), thrombocytosis (> 450 × 10^9^ cells/L), or thrombocytopenia (< 100 × 10^9^ cells/L) f) Autoimmune or chronic inflammatory pathology g) History or active solid or hematological malignancy h) Confirmed pancreatic insufficiency, chronic pancreatitis or previous pancreatic surgery i) ABI > 1.4 j) Taking immunosuppressive drugs, glucocorticoids or anticoagulants.

eGFR, estimated glomerular filtration rate; CKD-EPI equation: the Chronic Kidney Disease Epidemiology Collaboration equation; ABI, the ankle-brachial index.

The severity of PAD was assessed using the Fontaine classification, comprising four stages: I - asymptomatic; II - intermittent claudication; III - rest pain; and IV - ischemic ulcers or gangrene (28). Following the recommendations of the Inter-Society Consensus for the Management of Peripheral Arterial Disease (TASC II), patients with ischemic rest pain, ulcers, or gangrene, attributable to objectively proven PAD, were considered affected by critical limb ischemia (CLI) (29). Based on the T2DM – PAD patients’ clinical symptoms, the patients were divided into two groups, the first being those affected by stable PAD (Fontaine’s II) and the second being those affected by CLI (Fontaine’s III and IV).

### Demographic and clinical assessment

Data from the electronic medical records of the relevant departments was analyzed. Factors such as age, gender, diagnoses, and lab results were recorded. On the second hospital morning, blood samples were collected from all patients’ periphery. Laboratory personnel unaware of the patient’s diagnoses analyzed the blood samples.

The NHR, MHR, LHR, PHR, SII, SIRI, AISI, and triglyceride glucose index (TyG index) were calculated using the following formulas: NHR = neutrophil/HDL ratio; MHR = monocyte/HDL; LHR = lymphocyte/HDL ratio; PHR = platelet/HDL ratio; SII = platelet × neutrophil-to-lymphocyte ratio, SIRI = monocyte × neutrophil-to-lymphocyte ratio; AISI = neutrophil × platelet× monocyte-to-lymphocyte ratio; TyG index = Ln [Triglyceride (TG, mg/dl) × Fasting plasma glucose (mg/dl)/2].

### Statistical analysis

Statistical analyses were done using SPSS version 27.0 software (SPSS, Inc., Chicago, IL, United States). Graphs were created using Prism 9.0 (GraphPad Software). Continuous variables are described as means ± standard deviations (SDs) or medians (interquartile ranges), depending on the data distribution. Categorical variables are expressed as numbers and percentages of patients. Student’s t-test and the Mann-Whitney U test were used to compare the two groups. One-way ANOVA (normally distributed variables) or the Kruskal-Wallis test (non-normally distributed variables) was used to analyze the differences between the three groups. For categorical variables, chi-square tests were performed. Spearman’s correlation analysis was used to look at the connections between the different variables. Indicators with covariance were excluded from the correlation analysis. For example, NHR was covariant with neutrophils and HDL-C, and the Spearman correlation analysis was not conducted between the NHR and both variables. Binary logistic regression analysis was performed to explore the associations between NHR, MHR, PHR, LHR, SII, SIRI, and AISI and PAD. Due to the small variance of MHR as a continuous variable, the count unit was converted to x10^8^/mmol during logistic regression, then included in the regression model. The optimum value for identifying PAD risk in this sample was calculated using ROC curve analysis. The optimal cut-off value was determined by maximizing the Yoden index. A bilateral *P*< 0.05 was defined as statistically significant.

## Results

### Comparison of baseline clinical characteristics and laboratory indicators between the PAD group and WPAD group

The demographic and clinical features of T2DM-PAD group and T2DM-WPAD group are summarized in **Table 2**. Among the 434 diabetic patients enrolled, 218 had PAD, and 216 did not (WPAD). Compared to WPAD patients, PAD patients had a higher prevalence of coronary artery disease (CAD) and hypertension, and showed significantly increased levels of systolic blood pressure (SBP), HDL, low-density lipoprotein (LDL), C-reactive protein (CRP), neutrophils, lymphocytes, monocytes, NHR, MHR, PHR, SII, SIRI, and AISI(*P*<0.05). The two groups did not differ for gender, history of smoking, drinking, and dyslipidemia, diastolic blood pressure (DBP), fasting glucose, TyG index, HbA1c, TG, total cholesterol (TC), platelets, and LHR (*P*>0.05). The prevalence of Fontaine stage II, and CLI were 62.8, 12.9, and 11.5% in PAD patients, respectively.

**Table 2:** Demographic and clinical data of diabetic subjects with and without PAD.

Variables	WPAD	PAD	*P* value
	(N=216)	(N=218)	
Gender (male, %)	113 (52.3%)	132 (60.6%)	0.084
Age (years)	**56 (50-61.5)**	**65 (59-71)**	**<0.001**
Diabetes duration (years)	**4 (1-10)**	**10 (5-18)**	**<0.001**
Smoking, n (%)	73 (33.8%)	83 (38.1%)	0.353
Alcohol, n (%)	62 (28.7%)	71 (32.6%)	0.383
CAD (%)	**33 (15.3%)**	**63 (28.9%)**	**<0.001**
Hypertension, n (%)	**102 (47.2%)**	**142 (65.1%)**	**<0.001**
Dyslipidemia, n (%)	93 (43.1%)	92 (42.2%)	0.857
SBP (mmHg)	**126 (115-137)**	**133 (123-143)**	**<0.001**
DBP (mmHg)	80 (72-87)	78 (72-85)	0.135
Fasting glucose (mmol/l)	10.75 (7.53-15)	10.07 (7.65-14.71)	0.687
HbA1c (%)	8.3 (6.75-10.4)	8.1 (7.2-9.7)	0.842
TG, mmol/L	1.55 (1.08-2.24)	1.67 (1.14-2.48)	0.264
TC, mmol/L	4.63 (3.97-5.45)	4.01 (3.46-5.4)	0.104
HDL-C, mmol/L	**1.15 (0.98-1.39)**	**1.01 (0.88-1.15)**	**<0.001**
LDL-C, mmol/L	**2.93 (2.19-3.46)**	**2.52 (1.86-3.43)**	**0.016**
CRP, mg/L	**1.1 (0.65-2.1)**	**1.9 (1-4.3)**	**<0.001**
TyG index	7.84 (7.26-8.5)	7.97 (7.35-8.53)	0.629
Neutrophil,10^9^/L	**3.34 (2.83-4.13)**	**3.91 (3.2-4.86)**	**<0.001**
Lymphocyte, 10^9^/L	**1.71 (1.45-2.03)**	**1.47 (1.15-1.81)**	**<0.001**
Monocyte, 10^9^/L	**0.33 (0.27-0.4)**	**0.38 (0.3-0.48)**	**<0.001**
Platelet, 10^9^/L	206.5 (176-239.5)	200 (168-249)	0.653
NHR, 10^9^/mmol	**2.97 (2.62-3.72)**	**3.74 (3-5.26)**	**<0.001**
LHR, 10^9^/mmol	1.48 (1.17-1.82)	1.45 (1.1-1.97)	0.908
MHR, 10^9^/mmol	**0.29 (0.22-0.36)**	**0.37 (0.29-0.48)**	**<0.001**
PHR, 10^9^/mmol	**172.05 (141.98-222.18)**	**195.06 (158.16-260.61)**	**<0.001**
SII,10^9^/L	**409.56 (332.70-524.90)**	**534.59 (358.31-789.82)**	**<0.001**
SIRI,10^9^/L	**0.63 (0.47-0.89)**	**1.00 (0.68-1.46)**	**<0.001**
AISI,10^18^/L^2^	**129.46 (94.53-193.60)**	**195.56 (127.62-316.40)**	**<0.001**
PAD			
Fontaine’s II, n (%)		137 (62.8%)	/
CLI, n (%)		81 (37.2%)	/

CAD, coronary artery disease; SBP, systolic blood pressure; DBP, diastolic blood pressure; HbA1c, glycosylated hemoglobin; TG, triglyceride; TC, total cholesterol; HDL-C, high-density lipoprotein cholesterol; LDL-C, low-density lipoprotein cholesterol; CRP, C-reactive protein; TyG index, triglyceride glucose index; NHR, neutrophil/HDL-C ratio; LHR, lymphocyte/HDL-C ratio; MHR, monocyte/HDL-C ratio; PHR, platelet/HDL-C ratio; SII, systemic immune-inflammation index; SIRI, system inflammation response index; AISI, aggregate index of systemic inflammation. P <0.05 (two-sided) was defined as statistically significant. Bold values indicate statistically significance.

### Clinical and laboratory features of T2DM - PAD patients: subgroup analysis using the fontaine classification

The two groups did not differ regarding gender, age, duration of diabetes, history of CAD, hypertension, and dyslipidemia, SBP, DBP and laboratory parameters such as lymphocytes, monocytes, platelets, and LHR (*P*>0.05) (**Table 3**). As disease severity increased, fasting glucose, HbA1c, TG, TC, HDL-C, LDL-C, and TyG index presented a decreasing trend (*P*<0.05), but CRP, neutrophils, monocytes, NHR, MHR, PHR, SII, SIRI, and AISI showed an increasing trend (*P*<0.05). Fontaine stage II patients had higher percentage of smokers (44.5%) and alcoholics (40.1%) (*P*<0.05).

**Table 3:** Subgroup analysis of the clinical characteristics based on the Fontaine classification in patients with PAD.

Variables	Fontaine’s II	CLI	*P* value
	(N=137)	(N=81)	
Gender (male, %)	81 (59.1%)	51 (63%)	0.575
Age (years)	65 (59-69)	67 (60-72)	0.150
Diabetes duration (years)	10 (4.5-15)	11 (7-19)	0.066
Current smoker, n (%)	**61 (44.5%)**	**22 (27.2%)**	**0.011**
Alcohol, n (%)	**55 (40.1%)**	**16 (19.8%)**	**0.002**
CAD (%)	38 (27.7%)	25 (30.9%)	0.623
Hypertension, n (%)	89 (65%)	53 (52.8%)	0.944
Hyperlipidemia, n (%)	65 (47.4%)	27 (33.3%)	0.041
SBP (mmHg)	132 (120-143)	134 (125-144.5)	0.277
DBP (mmHg)	78 (70-85)	78 (73.5-86)	0.980
Fasting glucose (mmol/l)	**10.79 (8.47-15.61)**	**8.64 (6.54-11.795)**	**<0.001**
HbA1c (%)	**8.7 (7.2-10.25)**	**7.7 (7.15-8.85)**	**0.040**
TG, mmol/L	**1.81 (1.20-2.82)**	**1.45 (1.01-2.02)**	**0.004**
TC, mmol/L	**4.72 (3.895-5.52)**	**3.93 (3.285-5.12)**	**<0.001**
HDL-C, mmol/L	**1.05 (0.92-1.16)**	**0.96 (0.77-1.125)**	**0.003**
LDL-C, mmol/L	**2.59 (2.025-3.54)**	**2.32 (1.695-3.105)**	**0.019**
CRP, mg/L	**1.5 (0.9-3.35)**	**3 (1.5-11.1)**	**<0.001**
TyG index	**8.14 ± 0.92**	**7.65 ± 0.77**	**<0.001**
RBC (×10^9^/L)	**4.49 ± 0.49**	**3.99 ± 0.57**	**<0.001**
Neutrophil,10^9^/L	**3.7 (3.15-4.5)**	**4.18 (3.33-5.345)**	**0.021**
Lymphocyte, 10^9^/L	1.49 (1.125-1.855)	1.43 (1.16-1.76)	0.501
Monocyte, 10^9^/L	0.37 (0.285-0.45)	0.4 (0.31-0.5)	0.075
Platelet, 10^9^/L	200 (168-244.5)	212 (164.5-277)	0.349
NHR, 10^9^/mmol	**3.6 (2.93-4.6)**	**4.74 (3.16-6.02)**	**<0.001**
LHR, 10^9^/mmol	1.44 (1.09-1.86)	1.51 (1.13-2.2)	0.291
MHR, 10^9^/mmol	**0.34 (0.28-0.45)**	**0.41 (0.29-0.57)**	**0.003**
PHR, 10^9^/mmol	**192.11 (157.52-249)**	**200 (162.89-314.68)**	**0.032**
SII,10^9^/L	**504.44 (355.53-694.09)**	**600.32 (361.22-1027.89)**	**0.018**
SIRI,10^9^/L	**0.88 (0.64-1.31)**	**1.17 (0.77-1.73)**	**0.004**
AISI,10^18^/L^2^	**181.17 (121.82-276.4)**	**242.76 (141.15-429.44)**	**0.008**

CAD, coronary artery disease; SBP, systolic blood pressure; DBP, diastolic blood pressure; HbA1c, glycosylated hemoglobin; TG, triglyceride; TC, total cholesterol; HDL-C, high-density lipoprotein cholesterol; LDL-C, low-density lipoprotein cholesterol; CRP, C-reactive protein; TyG index, triglyceride glucose index; NHR, neutrophil/HDL-C ratio; LHR, lymphocyte/HDL-C ratio; MHR, monocyte/HDL-C ratio; PHR, platelet/HDL-C ratio; SII, systemic immune-inflammation index; SIRI, system inflammation response index; AISI, aggregate index of systemic inflammation. P <0.05 (two-sided) was defined as statistically significant. Bold values indicate statistically significance.

**Figure 1:**
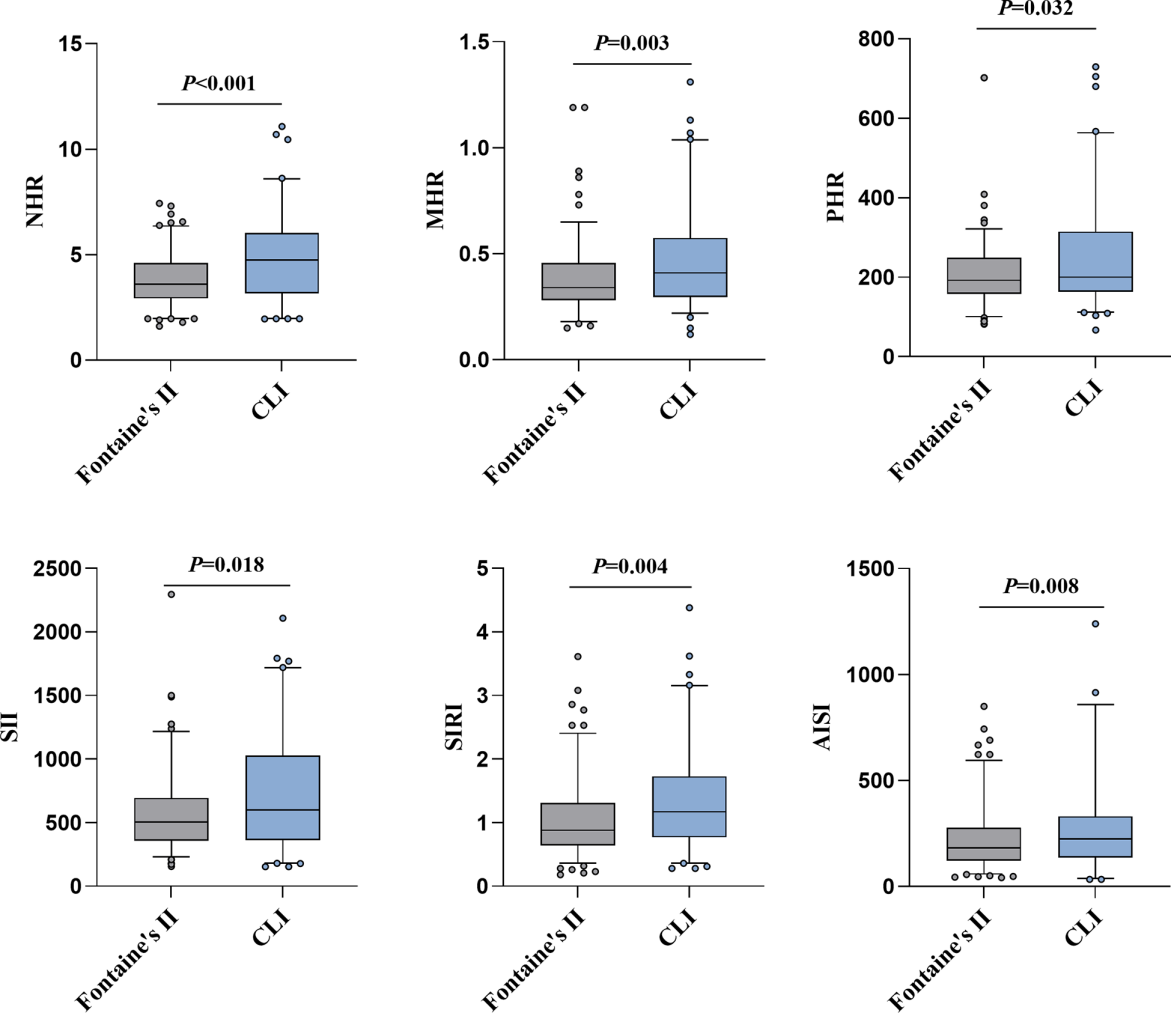
The NHR, MHR, PHR, SII, SIRI and AISI levels according to PAD severity. On the box plots, central lines represent the median, the length of the box represents the interquartile range and the lines extend to minimum and maximum values. Bold values indicate statistically significance.

The box - plot in **Figure 1** indicated that CLI patients had higher levels of NHR, MHR, PHR, SII, SIRI, and AISI, and that all these indices showed an increasing relationship with disease extent.

### Correlation of NHR, MHR, PHR, SII, SIRI, and AISI with other indicators of T2DM-PAD patients

Correlations between NHR, MHR, PHR, SII, SIRI, AISI and other indicators in PAD patients were assessed using Spearman correlation analysis. The NHR, MHR, PHR, SII, SIRI, and AISI were significantly correlated with Fontaine grading and CRP (*P*<0.05) (**Table 4**). However, these parameters had no significantly correlation with age, history of hypertension or dyslipidemia, SBP, DBP, fasting glucose, and TyG index. The PHR (r=-0.139) revealed a statistically weak connection with disease duration, whereas the NHR, MHR, SII, SIRI, and AISI were unrelated to disease duration. Additionally, a significant positive correlation was found between NHR and lymphocytes (r=0.151), monocytes (r=0.498), platelets (r=0.347), and TC (r=-0.207) (all *P*<0.05). The MHR was significantly linked with gender (r=-0.192), history of alcohol consumption (r=0.138), TC (r=-0.305), LDL-C (r=-0.192), neutrophils (r=0.455), lymphocytes (r=0.280), and platelets (r=0.204) (all *P*<0.05). The PHR was significantly associated with history of smoking (r=-0.136), alcohol consumption (r=-0.179) and CAD (r=-0.271), HbA1c (r=0.143), TG (r=0.169), TC (r=-0.133), neutrophils (r=0.270), and monocytes (r=0.182) (all *P*<0.05). The SII was significantly associated with monocytes (r=0.205) and the SIRI was significantly correlated with gender (r=-0.238) smoking history (r=0.167), TG (r=-0.141), and platelets (r=0.246) (all *P*<0.05).

**Table 4:** Correlation of the inflammatory biomarkers with other parameters in the T2DM-PAD patients.

	NHR		MHR		PHR		SII		SIRI		AISI	
	r	p	r	p	r	p	r	p	r	p	r	p
Gender	-0.068	0.316	**-0.192**	**0.005**	0.093	0.169	-0.073	0.282	-0.238	**<0.001**	-0.119	0.078
Age	-0.024	0.772	-0.079	0.245	-0.133	0.050	-0.054	0.431	-0.023	0.740	-0.078	0.249
Diabetes duration	-0.200	0.773	-0.089	0.189	**-0.139**	**0.041**	-0.061	0.374	-0.041	0.547	-0.034	0.618
Fontaine classification	**0.275**	**<0.001**	**0.240**	**<0.001**	**0.188**	**0.005**	**0.186**	**0.006**	**0.227**	**<0.001**	**0.209**	**0.002**
Smoking	0.057	0.402	0.122	0.073	**-0.136**	**0.045**	-0.001	0.989	0.167	**0.013**	0.045	0.508
Alcohol	-0.027	0.697	**0.138**	**0.042**	**-0.179**	**0.008**	-0.089	0.188	0.116	0.089	0.020	0.770
CAD	-0.059	0.384	-0.083	0.223	**-0.271**	**0.001**	**-0.148**	**0.029**	-0.041	0.543	-0.122	0.072
Hypertension	0.051	0.454	0.038	0.577	-0.039	0.565	-0.066	0.331	0.023	0.738	-0.029	0.674
Hyperlipidemia	0.008	0.903	-0.034	0.532	-0.054	0.431	-0.039	0.566	-0.002	0.974	-0.031	0.649
SBP	0.082	0.226	-0.011	0.875	0.025	0.710	0.131	0.053	0.109	0.109	0.095	0.164
DBP	0.092	0.175	0.107	0.115	0.062	0.366	-0.004	0.949	0.031	0.650	0.024	0.725
Fasting glucose	-0.003	0.967	-0.078	0.253	0.024	0.723	0.046	0.503	-0.035	0.604	-0.009	0.896
HbA1c	0.031	0.646	-0.013	0.848	**0.143**	**0.034**	0.065	0.338	-0.044	0.516	0.031	0.647
TG	0.333	0.632	-0.042	0.541	0.169	**0.012**	-0.071	0.299	**-0.141**	**0.038**	-0.059	0.384
TC	**-0.207**	**0.002**	-0.305	**<0.001**	-0.133	**0.049**	0.032	0.633	-0.071	0.297	-0.016	0.814
HDL-C	–	–	–	–	–	–	-0.047	0.494	-0.043	0.530	-0.064	0.345
LDL-C	-0.065	0.341	-0.192	**0.004**	-0.072	0.293	0.094	0.165	0.000	0.998	0.035	0.604
CRP	**0.497**	**<0.001**	0.379	**<0.001**	0.378	**<0.001**	0.364	**<0.001**	**0.360**	**<0.001**	0.375	**<0.001**
TyG index	0.012	0.855	-0.068	0.319	0.124	0.069	-0.031	0.651	-0.115	0.090	-0.050	0.459
Neutrophil	–	–	0.455	**<0.001**	0.27	**<0.001**	–	–	–	–	–	–
Lymphocyte	**0.151**	**0.026**	0.280	**<0.001**	0.117	0.085	–	–	–	–	–	–
Monocyte	**0.498**	**<0.001**	–	–	**0.182**	**0.007**	**0.205**	**0.002**	–	–	–	–
Platelet	**0.347**	**<0.001**	0.204	**0.002**	–	–	–	–	**0.246**	**<0.001**	–	–

CAD, coronary artery disease; SBP, systolic blood pressure; DBP, diastolic blood pressure; HbA1c, glycosylated hemoglobin; TG, triglyceride; TC, total cholesterol; HDL-C, high-density lipoprotein cholesterol; LDL-C, low-density lipoprotein cholesterol; CRP, C-reactive protein; TyG index, triglyceride glucose index; NHR, neutrophil/HDL-C ratio; LHR, lymphocyte/HDL-C ratio; MHR, monocyte/HDL-C ratio; PHR, platelet/HDL-C ratio; SII, systemic immune-inflammation index; SIRI, system inflammation response index; AISI, aggregate index of systemic inflammation. P <0.05 (two-sided) was defined as statistically significant. Bold values indicate statistically significance.

### Univariate and multivariate logistic regression analysis of the influencing factors for T2DM-PAD occurrence

The univariate logistic regression analysis showed that age, duration of diabetes, SBP, LDL-C, CRP, NHR, MHR, PHR, SII, SIRI, and AISI were independently associated with PAD occurrence in T2DM patients (**Table 5**). After excluding the effects of confounding factors for binary logistic regression, age, duration of diabetes, NHR, MHR, PHR, SII, SIRI, and AISI were still statistically significant and considered independent risk factors for PAD occurrence in T2DM patients.

**Table 5:** Univariate and binary logistic regression analysis results.

	Variable OR (95% CI)	P	Variable OR (95% CI)	P
Age	1.15(1.11-1.18)	<0.001	1.12(1.09-1.16)	<0.001
Diabetes duration	1.13(1.09-1.67)	<0.001	1.1(1.06-1.15)	<0.001
Hypertension	0.48(0.33-0.7)	<0.001		
SBP	1.02(1.01-1.03)	<0.001		
LDL-C	0.83(0.69-1)	0.047		
CRP	1.09(1.04-1.14)	<0.001		
NHR	1.83(1.55-2.16)	<0.001	1.78(1.43-2.19)	<0.001
MHR	1.66 (1.41-1.95)	<0.001	1.45 (1.14-1.83)	<0.001
PHR	1.005(1.003-1.007)	<0.001	1.008(1.004-1.011)	<0.001
SII	1.001(1.002-1.003)	<0.001	1.002(1.001-1.003)	<0.001
SIRI	5.31 (3.27-8.62)	<0.001	3.84(2.15-6.84)	<0.001
AISI	1.005(1.003-1.007)	<0.001	1.005(1.003-1.007)	<0.001

SBP, systolic blood pressure; LDL-C, low-density lipoprotein cholesterol; CRP, C-reactive protein; NHR, neutrophil/HDL-C ratio; MHR, monocyte/HDL-C ratio; PHR, platelet/HDL-C ratio; SII, systemic immune-inflammation index; SIRI, system inflammation response index; AISI, aggregate index of systemic inflammation. P <0.05 (two-sided) was defined as statistically significant.

### Diagnostic performance of different inflammatory indexes for T2DM-PAD

**Figure 2:**
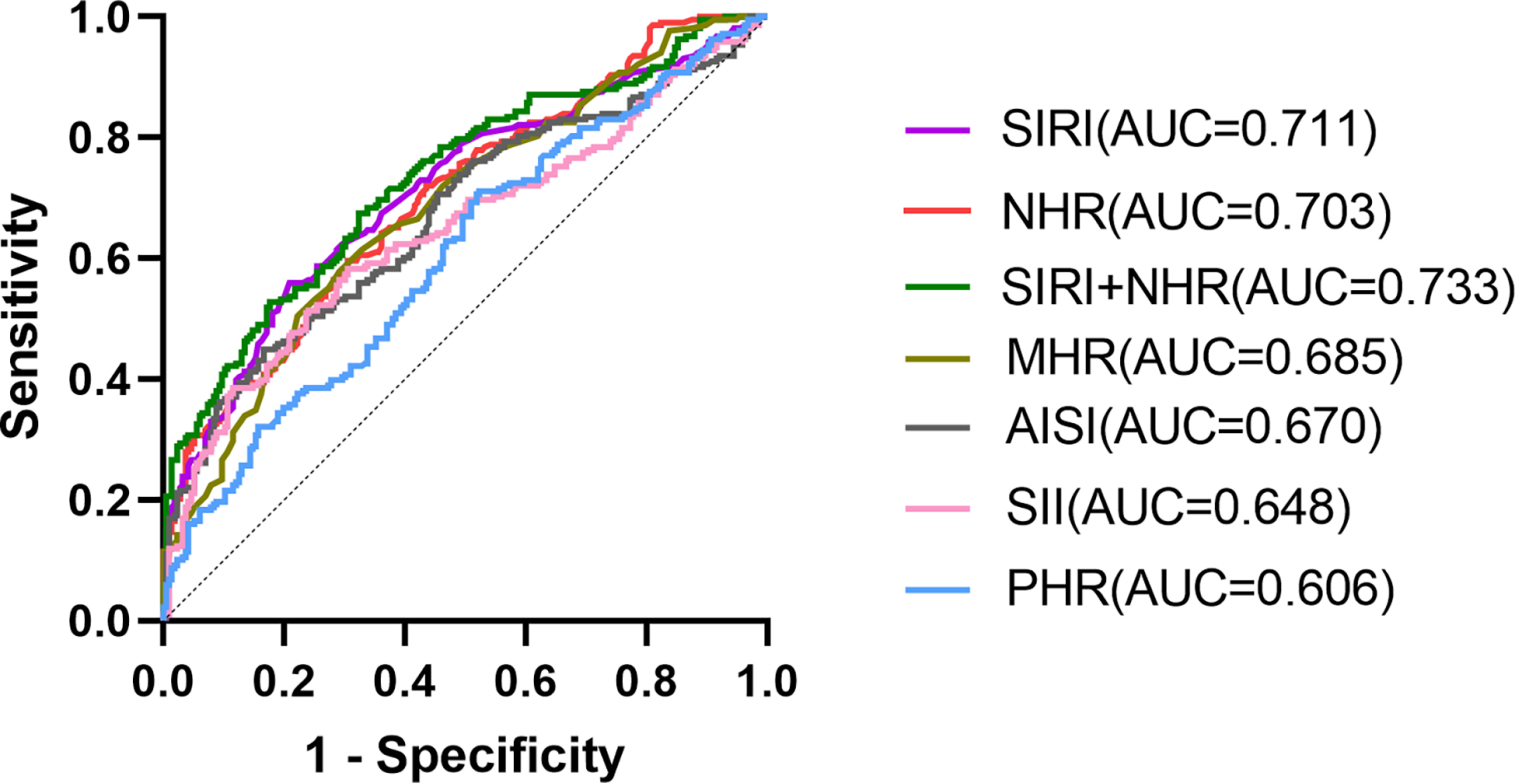
ROC curve analysis of the ability of these biomarkers to predict T2DM – PAD. SIRI (AUC 0.711, 95% CI:0.663–0.760, *P* = 0.000, cut-off 0.95, sensitivity 56%, specificity 79.2%); NHR (AUC 0.703, 95% CI:0.655–0.751, *P* = 0.000, cut-off 3.44, sensitivity 59.6%, specificity 69.4%); SIRI+NHR combined model(AUC 0.733, 95% CI:0.686-0.779, *P* = 0.000, cut-off 0.57, sensitivity 52.8%, specificity 82.4%); MHR(AUC 0.685, 95% CI:0.636–0.735, *P* = 0.000, cut-off 0.33, sensitivity 61.0%, specificity 70.4%);AISI(AUC 0.670, 95% CI:0.619–0.721, *P* = 0.000, cut-off 218.94, sensitivity45%, specificity83.3%);SII(AUC 0.648, 95% CI:0.596–0.700, *P* = 0.000, cut-off 486.96, sensitivity58.3%, specificity 69.4%);PHR (AUC 0.606, 95% CI:0.553–0.658, *P* = 0.000, cut-off 166.47, sensitivity71.1%, specificity 47.7%).

The ROC curve analysis was used to evaluate the ability of NHR, MHR, PHR, SII, SIRI, and AISI to identify T2DM-PAD patients. The results of the ROC curve analysis showed that each of these indicators exhibited a high discriminating value for T2DM-PAD. Both the SIRI (AUC 0.711, 95% CI: 0.663-0.760, *P* = 0.000, cut-off 0.95) and the NHR (AUC 0.703, 95% CI:0.655-0.751, *P* = 0.000, cut-off 3.44) had an AUC greater than 0.7 in the ROC analysis of T2DM-PAD. Additionally, the combination model of SIRI and NHR had an AUC of 0.733 (95% CI:0.686-0.779, *P* = 0.000, cut-off 0.57). Besides, the other indexes with AUCs greater than 0.6 were the MHR (AUC 0.685, 95% CI:0.636–0.735, *P* = 0.000, cut-off 0.33), AISI (AUC 0.670, 95% CI:0.619–0.721, *P* = 0.000, cut-off 218.94), SII (AUC 0.648, 95% CI:0.596–0.700, *P* = 0.000, cut-off 486.96) and PHR (AUC 0.606, 95% CI:0.553–0.658, *P* = 0.000, cut-off 166.47). The data are presented in **Figure 2**.

## Discussion

Recently, it has been demonstrated that NHR, MHR, LHR, PHR, SII, SIRI, and AISI are novel inflammatory biomarkers and have significant clinical value due to easy access. Nevertheless, no evidence exists on the relationship between these inflammatory parameters and T2DM - PAD. To our knowledge, there is no published research on the association between SIRI, AISI, and diabetic patients. In this retrospective cross-sectional study, the association was first explored between seven novel serological indicators and T2DM-PAD patients. This study illustrated that NHR, MHR, PHR, SII, SIRI, and AISI were strongly associated with increased PAD prevalence in T2DM patients, and that all these indices were associated with disease severity. Additionally, the ROC curve analysis showed that NHR, SIRI, and their combination might predict the T2DM-PAD occurrence more effectively than other indexes.

The daily life of patients is often severely affected by PAD, imposing a substantial medical expense on individuals and society. Therefore, effective early screening and identification of T2DM - PAD patients is crucial (30). Evidence suggests that diabetes is one of the strongest risk factors for PAD development (31). Hence, only diabetic patients were chosen to be investigated in this study. Several potential biomarkers have been identified for PAD in diabetic patients, including HMGB 1, OPG, FGF 23, Omentin-1, Cyr61, and Sortilin (32–36). However, these biomarkers require complex and costly measurements and are not widely used in the clinic. The new inflammatory indicators presented in this work can be easily obtained using standard laboratory indices and may have substantial clinical use.

It is generally agreed that atherosclerosis is a chronic inflammatory disease of the arterial wall that stems from an insufficient inflammatory response and an imbalanced lipid metabolism. The role of inflammation and lipid metabolism in T2DM and PAD pathogenesis is of great interest (37, 38). Monocytes initiate and promote atherosclerosis progression by releasing pro-inflammatory cytokines, reactive oxygen species, and protein hydrolases (39). Neutrophils, the most abundant leukocyte subtype, exacerbate vessel wall inflammation *via* apoptosis of small muscle cells (40, 41). In contrast, lymphocytes can impair atherosclerosis progression (36). Platelets have a dual role in atherosclerosis: their adherence to the vascular wall promotes plaque formation (42), whereas their activation promotes inflammation and thrombosis (43). Elevated LDL and reduced HDL levels are key factors in atherosclerosis development and progression (44). The current results showed that PAD patients had significantly higher neutrophils, monocytes, and CRP and significantly fewer lymphocytes and HDL than WPAD patients, as well as a significant reduction in LDL, which could be hypothesized might be related to statin use. However, statin use was not an exclusion criterion, and the **supplementary tables** showed that the two groups of patients using statins had inflammatory indicators that were not significantly different (See **Supplementary Tables 1**, **2**).

The MHR, NHR, PHR, and LHR have been investigated as novel inflammatory markers derived from peripheral blood cells and HDL-C in many systemic chronic inflammatory diseases. The SII, SIRI, and AISI are novel chronic low-grade inflammatory markers based on peripheral blood cells and platelets. The current results showed that NHR, MHR, PHR, SII, SIRI, and AISI were significantly higher in T2DM-PAD patients in WPAD patients and that they were significantly correlated with disease severity based on the Fontaine classification. Based on the ultrasound results of the lower limbs, we evaluated the degree of PAD disease and then categorized the patients with T2DM - PAD into three subgroups: mild, moderate, and severe. Individuals with severe PAD had higher concentrations of NHR, MHR, PHR, SII, SIRI, and AISI than those with mild PAD. Significant incremental increases were not reflected in the three sub-periods (See **Supplementary Figure 1**). These results were consistent with L. Santoro et al. that MHR was not associated with ultrasound grading in patients with only PAD (24), suggesting that these inflammatory indicators might be more appropriate for clinical application in combination with the Fontaine classification to assess disease severity. Spearman analysis showed that all indicators were positively associated with the Fontaine classification and CRP. Meanwhile, NHR, MHR, SII, SIRI, and AISI were not correlated with the patients’ age and disease duration, and PHR was weakly negatively correlated with the duration of diabetes. Although high glucose and insulin resistance enhance vascular inflammation (45), the data showed no significant correlation between these inflammatory markers and fasting glucose in T2DM-PAD patients or the total population, which might be due to sample size limits. The TyG index, a surrogate for insulin resistance, is significantly related to the gold standard hyperinsulinemic-orthoglycemic clamp (46) and can be a reliable assessment of insulin resistance in patients. Unfortunately, no correlation between TyG index and these indices was observed in the T2DM-PAD population. But in the total population, NHR (r=0.115), MHR (r=0.095), PHR (r=0.156) were found to be significantly correlated with TyG index (all P<0.05) (See **Supplementary Table 3**). Further large cohort studies are needed to analyze the relationship of these indicators with glycemic and insulin resistance. The univariate logistic regression showed that age, duration of diabetes, history of hypertension, SBP, CRP, NHR, MHR, PHR, SII, SIRI, and AISI were statistically significant. The multifactorial regression, excluding the effects of confounding factors, showed that NHR, MHR, PHR, SII, SIRI, and AISI were independently associated with T2DM-PAD. This study also demonstrated that age and duration of diabetes might be independent risk factors, consistent with previous studies (30). Combined with the Spearman correlation results, it was hypothesized that disease prediction by NHR, MHR, SII, SIRI, and AISI was not affected by age and duration of diabetes, increasing their clinical adjunctive value, but this hypothesis might require prospective cohorts to verify reliability. The ROC curve analysis showed that NHR, MHR, PHR, SII, SIRI, and AISI could predict T2DM-PAD well. The AUCs of MHR, AISI, SII, and PHR were over 0.6, and NHR and SIRI were over 0.7. The highest AUC (0.733) was detected when the NHR and SIRI were combined, indicating that it was better to use their combination for disease prediction in the clinic. When the combined model of NHR and SIRI value was greater than 0.57, it might suggest that the patient had a higher risk of developing PAD. This finding would serve as an easily available diagnostic aid for clinicians.

However, this current study also has some limitations. First, this was a retrospective cross-sectional study conducted in a single center, unable to determine the causal relationship between disease and indicators. Although these indices showed a good correlation with PAD, further studies are necessary to consider them as independent risk factors for the disease. Second, this research did not exclude patients on statins because neither group had significant differences in emerging inflammatory indicators on statins. Third, all patients’ body mass index (BMI) data were not collected completely and BMI was not included in the analysis, so the possible effect of BMI as a confounding factor may had been overlooked. Fourth, the novel inflammatory indicators were not dynamically monitored. Therefore, whether their changes are related to PAD progression remains unknown. Further prospective studies are required to analyze whether the above indicators reduce atherosclerosis progression. SIRI effectively predicts MACE in patients undergoing percutaneous coronary intervention after acute coronary syndrome (14), and this predictive performance exceeds the neutrophil-lymphocyte ratio (NLR) and monocyte-lymphocyte ratio (MLR) (47). Prospective studies are needed to see if these indices have a similar effect in predicting MACE and MALE for PAD.

## Data availability statement

The raw data supporting the conclusions of this article will be made available by the authors, without undue reservation.

## Ethics Statement

The study protocol was approved by the ethics committee of the Liyuan Hospital, Tongji Medical College, Huazhong University ofScience and Technology. (Approval IRBID: [2022] IEC CRYJ 0018). All data used in this study were anonymized and the requirement forinformed consent was waived.

## Author contributions

SJ and YiS conceived the study plan and contributed to the revision of the final manuscript. YiS collected, analyzed the data and finished the manuscript writing. YZ, YaS and LZ participated in data collection and literature search. WC, LW, MS, BX, RW, ZF, YY and FY contributed to the manuscript writing and data interpretation. All authors contributed to the article and approved the submitted version.
