# Supplementary material for: Combination model of neutrophil to high-density lipoprotein ratio and system inflammation response index is more valuable for predicting peripheral arterial disease in type 2 diabetic patients: A cross-sectional study
Source: Front Endocrinol (Lausanne). 2023 Feb 16;14:1100453. doi: 10.3389/fendo.2023.1100453 (PMC9978802; doi:10.3389/fendo.2023.1100453)
Supplement: Supplementary file 1 [file DataSheet_1.docx]

Supplementary Material

**Method**

ABI ⩽ 0.9 was taken as the diagnostic criteria for the presence of PAD. Patients diagnosed as PAD had limb extremity arteries examined by arterial Doppler-enhanced ultrasonography. The examination sites were the common femoral artery, femoral artery bifurcation, popliteal artery, posterior tibial artery and dorsalis pedis artery. The vascular pathology was evaluated and scored as follows: (1) Artery intima thickness: normal (<1 mm), 0 point; mildly thickened (1–1.2 mm), 1 point; severely thickened (>1.2 mm), 2 points. (2) Hardening: normal, 0 point; mildly hardened (the intima was not thickened, the echo was increased, and without plaque), 1 point; moderately to severely hardened (mildly hardened, associated with plaque or stenosis), 2 points. (3) Plaque: normal (no plaque formation), 0 point; single plaque, 1 point; multiple plaques, 2 points; diffused plaques, 3 points. (4) Stenosis: normal, 0 point; mild stenosis (narrowing by 30%–50%), 1 point; moderate or severe stenosis (narrowing by 50%–75%), 2 points; occlusion (no blood flow), 3 points. The severity of PAD was classified according to the total points: (1) 0 point, normal; (2) <10 mild; (3) 10–20 points, moderate; (4) >20 points, severe (1).

We separated T2DM-PAD and T2DM-WPAD patients based on whether or not they were taking statins to determine whether statin use affected our inflammatory index levels. As shown in the Supplementary Table 1 and Table 2, there were no significant differences between the statin and non-statin subgroups in terms of NHR, MHR, PHR, LHR, SII, SIRI, and AISI.

Based on the ultrasound results of the lower limbs, we evaluated the degree of PAD disease and then categorized the patients with T2DM - PAD into three subgroups: mild, moderate, and severe. According to the Supplementary Figure 1, individuals with severe PAD had higher concentrations of NHR, MHR, PHR, SII, SIRI, and AISI than those with mild PAD. There was no significant correlation between these inflammatory markers and disease severity in any of the three patient groups.

In the T2DM-PAD population, no correlation of these indicators with glucose and TyG index was observed. However, in the total population, NHR (r=0.115), MHR (r=0.095), PHR (r=0.156) were found to be significantly correlated with TyG index (all P<0.05) (See Supplementary Table 3).

**Supplementary Table 1. Baseline and laboratory findings of T2DM - WPAD patients with statins or without statins**

| Variables | Non-statins | statins | *P* value |
| --- | --- | --- | --- |
|  | (N=192) | (N=24) |  |
| Gender (male，%） | 101（52.6%） | 12（50%） | 0.81 |
| Age (years) | **55±8.79** | **57±9.24** | **0.049** |
| Diabetes duration (years) | 4（1-10） | 5（2.5-10） | 0.29 |
| Smoking, n (%) | 65（33.9%） | 8（33.3%） | 0.959 |
| Alcohol, n (%) | 56（29.2%） | 6（25%） | 0.671 |
| CAD (%) | **23（12%）** | **10（41.7%）** | **＜0.001** |
| Hypertension, n (%) | **84（43.8%）** | **18（75%）** | **0.004** |
| Dyslipidemia, n (%) | **72（37.5%）** | **21（87.5%）** | **＜0.001** |
| SBP (mmHg) | 127±17.18 | 121±14.15 | 0.932 |
| DBP (mmHg) | **80（72-88）** | **76（68-81）** | **0.015** |
| Fasting glucose (mmol/l) | **11.17（7.71-15.06）** | **7.61（6.17-11.86）** | **0.005** |
| HbA1c (%) | **8.45（6.90-10.55）** | **7.20（6.50-8.45）** | **0.026** |
| TG, mmol/L | 1.55（1.09-2.37） | 1.34（0.99-1.85） | 0.221 |
| TC, mmol/L | **4.83±1.17** | **3.84±0.90** | **＜0.001** |
| HDL-C, mmol/L | 1.14（0.98-1.41） | 1.18（1.01-1.38） | 0.899 |
| LDL-C, mmol/L | **3.03±0.98** | **1.98±0.71** | **＜0.001** |
| CRP, mg/L | 1.10（0.70-2.20） | 0.85（0.50-1.85） | 0.281 |
| TyG index | **7.92(7.32-8.54)** | **7.51(6.97-7.84)** | **0.020** |
| Neutrophil ,10^9^/L | 3.36（2.83-4.20） | 3.21（2.81-3.70） | 0.456 |
| Lymphocyte, 10^9^/L | 1.72（1.53-2.04） | 1.63（1.34-1.88） | 0.202 |
| Monocyte, 10^9^/L | 0.33（0.27-0.40） | 0.37（0.26-0.40） | 0.666 |
| Platelet, 10^9^/L | 207（176-239） | 206（190-249） | 0.454 |
| NHR, 10^9^/mmol | 2.98±1.14 | 2.89±1.08 | 0.782 |
| LHR, 10^9^/mmol | 1.50（1.18-1.83） | 1.33（1.06-1.72） | 0.39 |
| MHR, 10^9^/mmol | 0.29（0.22-0.36） | 0.29（0.23-0.35） | 0.803 |
| PHR, 10^9^/mmol | 171.78（141.47-222.07） | 180.03（142.51-233.33） | 0.651 |
| SII,10^9^/L | 409.56（328.50-519.83） | 412.03（334.82-563.73） | 0.747 |
| SIRI,10^9^/L | 0.63（0.46-0.88） | 0.66（0.52-0.95） | 0.513 |
| AISI,10^18^/L^2^ | 125.94（94.12-193.60） | 145.27（104.56-199.70） | 0.475 |
| NLR | 1.98(1.58-2.41) | 1.97(1.47-2.70) | 0.872 |
| PLR | 114.53(97.59-145.32) | 134.81(100.15-155.79) | 0.155 |
| MLR | 0.18(0.16-0.24) | 0.21(0.16-0.27) | 0.186 |

CAD, coronary artery disease; SBP, systolic blood pressure; DBP, diastolic blood pressure; HbA1c, glycosylated hemoglobin; TG, triglyceride; TC, total cholesterol; HDL-C, high-density lipoprotein cholesterol; LDL-C, low-density lipoprotein cholesterol; CRP, C-reactive protein; TyG index, triglyceride glucose index; NHR, neutrophil/HDL-C ratio; LHR, lymphocyte/HDL-C ratio; MHR, monocyte/HDL-C ratio; PHR, platelet/HDL-C ratio; SII, systemic immune-inflammation index; SIRI, system inflammation response index; AISI, aggregate index of systemic inflammation; NLR, neutrophil-lymphocyte ratio; PLR, platelet-lymphocyte ratio; MLR, monocyte-lymphocyte ratio. *P* <0.05 (two-sided) was defined as statistically significant. Bold values indicate statistically significance.

**Supplementary Table 2. Baseline and laboratory findings of T2DM - PAD patients with statins or without statins**

| Variables | Non-statins | statins | *P* value |
| --- | --- | --- | --- |
|  | (N=174) | (N=44) |  |
| Gender (male，%） | 108（62.1%） | 24（54.5%） | 0.362 |
| Age (years) | 64±7.7 | 67±6.5 | 0.049 |
| Diabetes duration (years) | 10（5-16） | 11（7-20） | 0.146 |
| Smoking, n (%) | 68（39.1%） | 15（34.1） | 0.543 |
| Alcohol, n (%) | 58（33.3%） | 13（29.5%） | 0.632 |
| CAD (%) | **43（24.7%）** | **20（45.5%）** | **0.007** |
| Hypertension, n (%) | **107（61.5%）** | **35（79.5%）** | **0.025** |
| Dyslipidemia, n (%) | **54（31%）** | **38（86.3%）** | **＜0.001** |
| SBP (mmHg) | 133（122-143） | 134（124-144） | 0.833 |
| DBP (mmHg) | 78±10.2 | 77±10.6 | 0.386 |
| Fasting glucose (mmol/l) | 10.07（7.95-15.19） | 9.92（6.93-13.64） | 0.258 |
| HbA1c (%) | 8.3(7.2-10.1) | 8.0(7.2-9.1) | 0.472 |
| TG, mmol/L | 1.74（1.14-2.57） | 1.49（1.14-2.03） | 0.202 |
| TC, mmol/L | **4.71（3.73-5.54）** | **3.67(3.27-4.29)** | **＜0.001** |
| HDL-C, mmol/L | 1.00(0.88-1.15) | 1.02(0.88-1.22) | 0.515 |
| LDL-C, mmol/L | **2.72(2.09-3.61)** | **1.92(1.63-2.35)** | **＜0.001** |
| CRP, mg/L | 2.0(1.0-4.3) | 1.60(0.85-3.85) | 0.198 |
| TyG index | 8±0.9 | 7.79±0.88 | 0.158 |
| Neutrophil ,10^9^/L | 3.89(3.11-4.90) | 3.94(3.37-4.78) | 0.505 |
| Lymphocyte, 10^9^/L | 1.47(1.13-1.79) | 1.47(1.20-1.84) | 0.838 |
| Monocyte, 10^9^/L | 0.38(0.30-0.48) | 0.37(0.30-0.50) | 0.828 |
| Platelet, 10^9^/L | 206(170-251) | 189(156-243) | 0.246 |
| NHR, 10^9^/mmol | 3.74（3.00-5.24） | 3.81（3.00-5.27） | 0.769 |
| LHR, 10^9^/mmol | 1.46(1.10-1.97) | 1.44(1.14-1.93) | 0.794 |
| MHR, 10^9^/mmol | 0.37（0.29-0.48） | 0.37（0.28-0.51） | 0.95 |
| PHR, 10^9^/mmol | 197.66（159.41-256.70） | 191.95（143.21-271.25） | 0.503 |
| SII,10^9^/L | 536.53（363.03-792.78） | 525.81（327.32-725.97） | 0.7 |
| SIRI,10^9^/L | 1.01（0.68-1.43） | 0.94（0.68-1.52） | 0.854 |
| AISI,10^18^/L^2^ | 196.53（129.71-323.03） | 161.24（122.42-299.46） | 0.736 |
| NLR | 2.57(1.9-3.64) | 2.76(1.92-3.56) | 0.780 |
| PLR | 136.96(103.65-183.65) | 131.74(84.73-199.98) | 0.521 |
| MLR | 0.26(0.2-0.35) | 0.24(0.2-0.33) | 0.757 |

CAD, coronary artery disease; SBP, systolic blood pressure; DBP, diastolic blood pressure; HbA1c, glycosylated hemoglobin; TG, triglyceride; TC, total cholesterol; HDL-C, high-density lipoprotein cholesterol; LDL-C, low-density lipoprotein cholesterol; CRP, C-reactive protein; TyG index, triglyceride glucose index; NHR, neutrophil/HDL-C ratio; LHR, lymphocyte/HDL-C ratio; MHR, monocyte/HDL-C ratio; PHR, platelet/HDL-C ratio; SII, systemic immune-inflammation index; SIRI, system inflammation response index; AISI, aggregate index of systemic inflammation; NLR, neutrophil-lymphocyte ratio; PLR, platelet-lymphocyte ratio; MLR, monocyte-lymphocyte ratio. *P* <0.05 (two-sided) was defined as statistically significant. Bold values indicate statistically significance.

**Supplementary Figure 1. The inflammatory biomarkers’ levels according to PAD severity based on ultrasound results.**
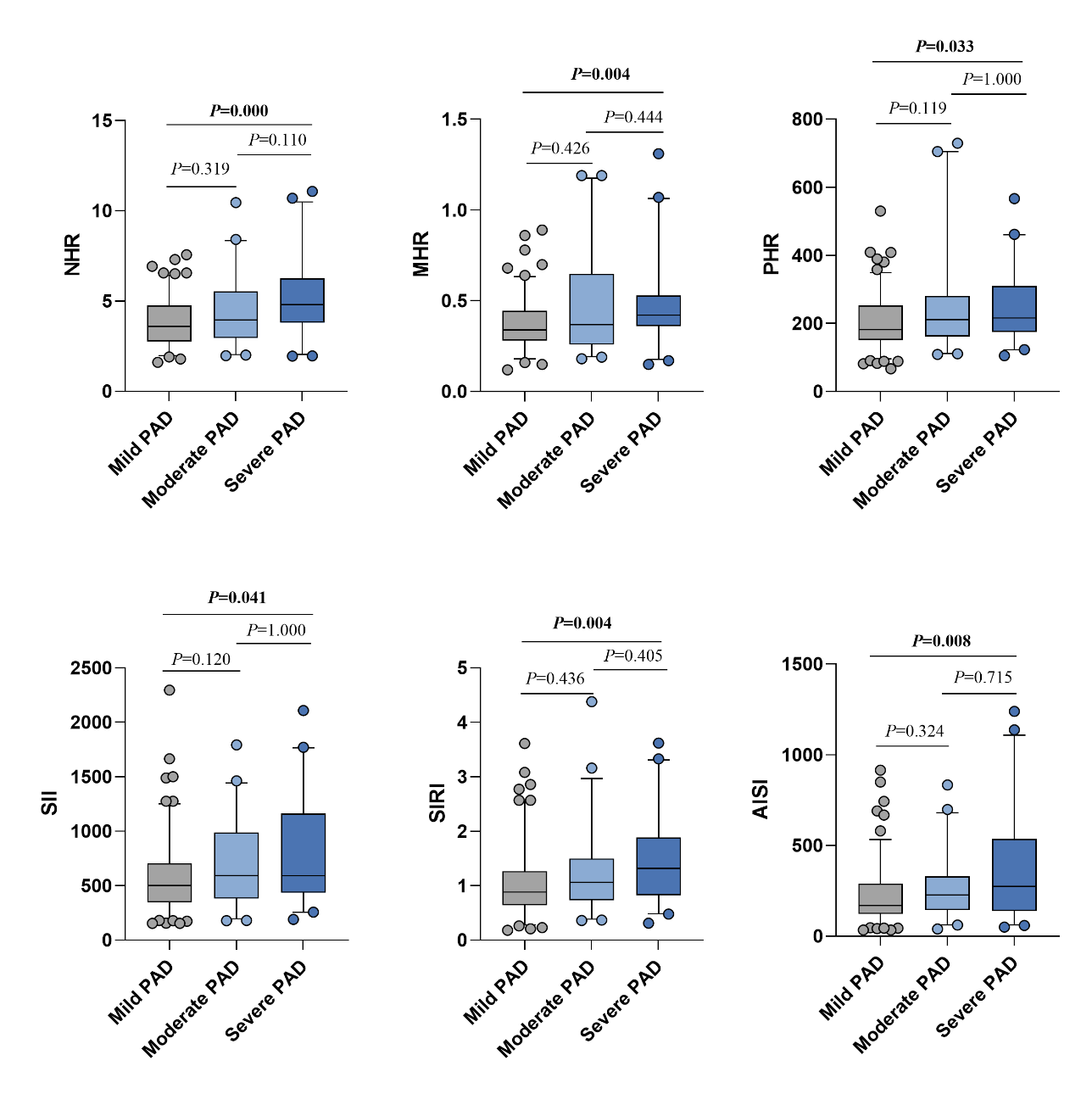


On the box plots, central lines represent the median, the length of the box represents the interquartile range and the lines extend to minimum and maximum values. *P* <0.05 (two-sided) was defined as statistically significant. Bold values indicate statistically significance.

**Supplementary Table 3. Correlation of the inflammatory biomarkers with Fasting glucose, HbA1c and TyG index in the whole patients.**

HbA1c, glycosylated hemoglobin; TyG index, triglyceride glucose index. *P* <0.05 (two-sided) was defined as statistically significant. Bold values indicate statistically significance.

**Reference:**

1. Jiang, W.; Tang, Q.; Zhang, L.; Chao, Y.; Hong, B. Association of Serum C1q Tumour Necrosis Factor–Related Protein 9 with the Severity of Lower Extremity Peripheral Arterial Disease in Type 2 Diabetes Patients. Diabetes and Vascular Disease Research 2018, 15 (3), 270–273.
